# Schistosomiasis as a Cause of Chronic Lower Abdominal Pain

**DOI:** 10.1155/S1064744995000093

**Published:** 1995

**Authors:** Kathir G. Yoganathan, Tom J. McManus

**Affiliations:** 1 Department of Genito-Urinary Medicine Singleton Hospital Wales Swansea SA2 8QA United Kingdom; 2 Department of Genito-Urinary Medicine King's College Hospital London United Kingdom

## Abstract

*Background:* Chronic intestinal schistosomiasis is rare in the United Kingdom. The symptoms are nonspecific and may mimic several other gastrointestinal conditions. We present a case of chronic intestinal schistosomiasis in a West Indian woman presenting to a genitourinary clinic.

*Case:* The patient presented with chronic lower abdominal pain and dysuria. A sexually transmitted disease (STD) screen was negative and midstream urine cultures were sterile. A rectal biopsy revealed a non-necrotizing granulomatous reaction around the ova of Schistosoma. Her symptoms resolved with anti-schistosomiasis therapy.

*Conclusion:* This case illustrates that physicians should be aware of chronic schistosomiasis in the differential diagnosis of chronic lower abdominal pain in women who have come from or visited areas where schistosomiasis is endemic.


Infectious Diseases in Obstetrics and Gynecology 2:235–238 (1995)
© 1995 Wiley-Liss, Inc.

### Schistosomiasis as a Cause of Chronic Lower Abdominal Pain

**Kathir G. Yoganathan and Tom J. McManus**

*Department of Genito-Urinary Medicine, Singleton Hospital, Swansea, Wales (K.G.Y.), and Department of Genito-Urinary Medicine, King's College Hospital, London, United Kingdom (T.J.M.)*

Abstract

***Background:*** **Chronic intestinal schistosomiasis is rare in the United Kingdom. The symptoms are nonspecific and may mimic several other gastrointestinal conditions. We present a case of chronic intestinal schistosomiasis in a West Indian woman presenting to a genitourinary clinic.**

***Case:*** **The patient presented with chronic lower abdominal pain and dysuria. A sexually transmitted disease (STD) screen was negative and midstream urine cultures were sterile. A rectal biopsy revealed a non-necrotizing granulomatous reaction around the ova of Schistosoma. Her symptoms resolved with anti-schistosomiasis therapy.**

***Conclusion:*** **This case illustrates that physicians should be aware of chronic schistosomiasis in the differential diagnosis of chronic lower abdominal pain in women who have come from or visited areas where schistosomiasis is endemic.** © 1995 Wiley-Liss, Inc.

Key words

Pelvic inflammatory disease, urinary tract infection, rectal biopsy

We report a case of chronic intestinal schistosomiasis in a woman attending a department of genitourinary medicine in London. The condition is extremely rare in the United Kingdom and, if the physician is unfamiliar with the clinical presentation of schistosomiasis, it can be misdiagnosed as pelvic inflammatory disease. We suggest that chronic schistosomiasis should be included in the differential diagnosis of chronic lower abdominal pain if the patient has lived in or visited a high-risk area.

### CASE REPORT

A 37-year-old woman first presented to the Department of Genito-Urinary Medicine at King's College Hospital, London, in November 1990 with a 3-month history of frequency, dysuria, and abdominal pain which she described as a constant dull ache. She had been sterilized 10 years previously and had not had sexual intercourse for 2 years. The patient, who was born in St. Lucia, had emigrated to the United Kingdom at the age of 10 years. She had visited St. Lucia for holidays on 3 occasions during the previous 10 years. Her most recent foreign travel had been to Israel in 1986. She could not recall ever having swum in fresh water. The examination was unremarkable apart from a palpable colon. A full sexually transmitted disease (STD) screen which included serological tests for syphilis; cervical tests for *Chlamydia trachomatis* [enzyme immunosorbent assay (EIA)] and *Neisseria gonorrhoeae* (culture); and vaginal swabs for *Trichomonas vaginalis*, *Candida* species (microscopy and culture), and anaerobic (bacterial) vaginosis (microscopy) was negative. The cervical cytology showed mild dyskaryosis. The patient was treated for a presumptive urinary tract infection but a subsequent midstream urine culture was reported as sterile. She

**Address correspondence/reprint requests to Dr. Kathir G. Yoganathan, Department of Genito-Urinary Medicine, Singleton Hospital, Swansea SA2 8QA, Wales, United Kingdom.**

Received August 5, 1994
Accepted December 27, 1994

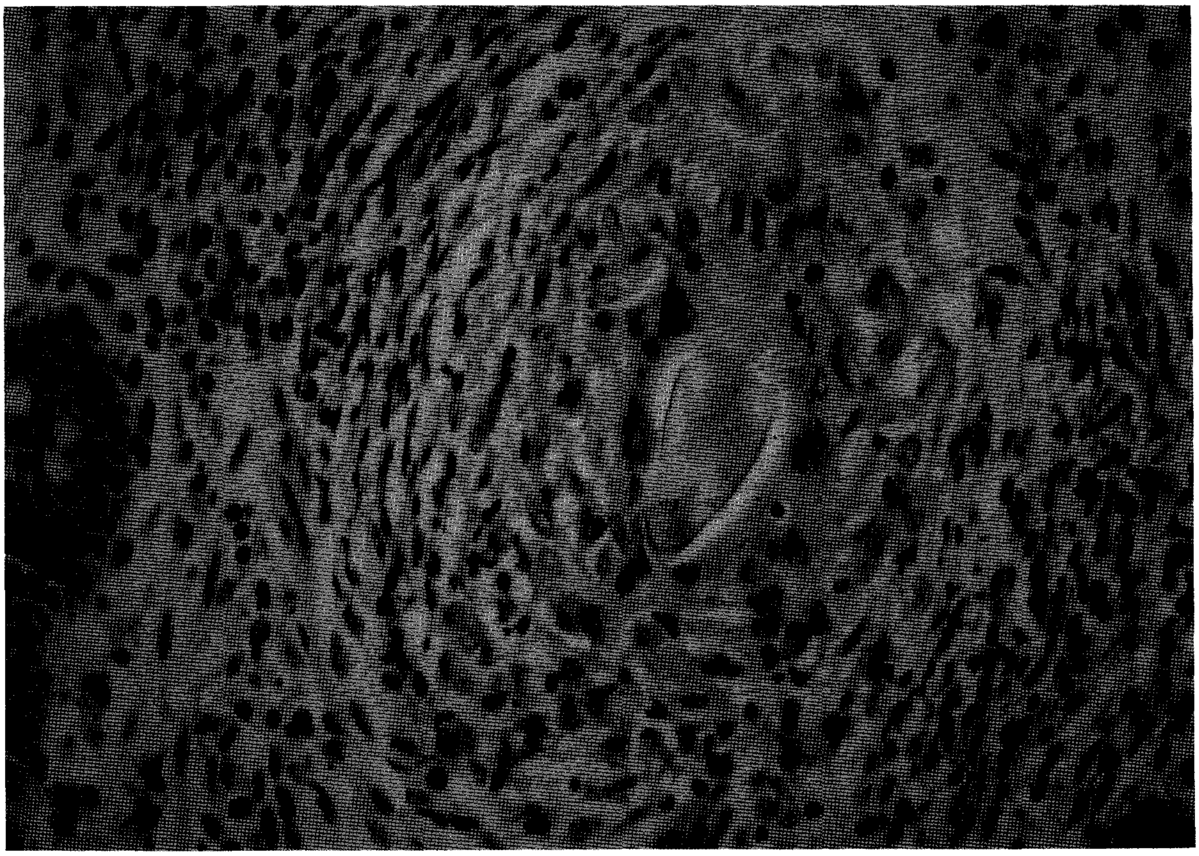

Fig. 1. Rectal biopsy showing granulomatous reaction around the ova of *Schistosoma*.

failed to appear for a follow-up examination but 6 months later returned with symptomatic vulvovaginal candidiasis which was treated successfully with clotrimazole pessaries and cream. A second full STD screen was also negative, and repeat cervical cytology showed no abnormality. One month later, she returned complaining of persistent lower abdominal pain, back pain, and frequency of micturition. She was afebrile. The clinical diagnosis of pelvic inflammatory disease was made on the basis of lower abdominal tenderness and pain on manipulation of the cervix. She was treated with doxycycline (a 200-mg single dose followed by 100 mg daily for 10 days) and metronidazole (400 mg b.i.d. for 5 days). Following treatment, the abdominal pain persisted. On direct questioning, the patient admitted passing a small quantity of mucus from the rectum. A rectal examination was normal, but sigmoidoscopy showed an erythematous, friable mucosa. A rectal biopsy revealed a non-necrotizing granulomatous reaction around the ova of *Schistosoma* (Fig. 1). A barium enema showed superficial ulceration limited to the rectum and sigmoid colon (Fig. 2).

A microscopy of terminal urine was negative. Routine biochemistry and hematology including eosinophil counts were normal. A schistosomal ELISA test was positive at a significant level. *Strongyloides* serology was negative and *Entamoeba histolytica* cysts were found incidentally on a stool parasite screen. Radiology of the renal tract was normal. The schistosomal infection was treated with praziquantel, 20 mg/kg b.i.d. for 3 days, and diloxanide furoate, 500 mg t.i.d. for 10 days, to eradicate the intestinal amoebic cysts. This treatment resulted in complete resolution of the patient's urinary and abdominal symptoms. Two months after treatment, a follow-up sigmoidoscopy showed a normal mucosa and a rectal snip was negative for *Schistosoma* ova.

### DISCUSSION

The 3 principal species of *Schistosoma* affecting humans are *S. mansoni*, *S. haematobium*, and *S.*

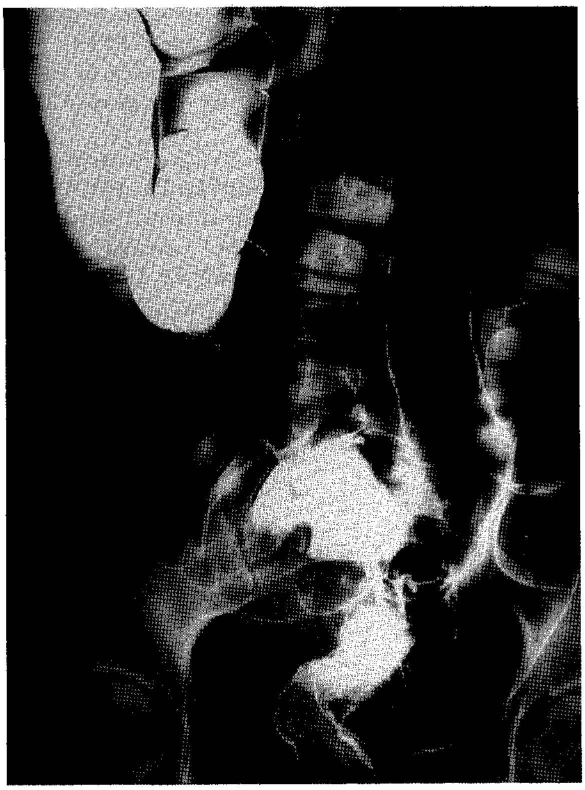

**Fig. 2. Barium enema showing mucosal irregularity and superficial ulceration in the rectum and sigmoid colon.**

*japonicum*. Globally, the most widespread infection is *S. mansoni*, which is also found in parts of the Caribbean islands including St. Lucia.[1] *S. mansoni* commonly causes disease of the large intestine,[2] with the viable ova being the antigenic stimulus[3] that produces an inflammatory reaction, granuloma formation, papillomas, ulceration, bleeding, and subsequently fibrosis formation giving rise to the long-term sequelae of the disease.[4] The symptoms of colonic schistosomiasis are often nonspecific and may mimic several other gastrointestinal pathologies. Mohamed et al.[4] reported that nonspecific abdominal pain was the most common symptom in colonic schistosomiasis. Other common symptoms are diarrhea, rectal bleeding, and alternating diarrhea and constipation.[5] Our patient presented with lower abdominal pain, passage of mucus through the rectum, and a palpable colon. In the female, the pelvic organs are a common site for ectopic schistosomiasis. Other reported sites of infection include the spinal cord,[6] lung, testis, brain, and duodenum.[7] Our patient had had recurrent symptoms suggestive of pelvic inflammatory disease, and it is possible that she may have had involvement of the fallopian tubes causing bilateral adnexal tenderness. However, fallopian-tube involvement is relatively uncommon,[8] the cervix being the most common site of infection in the female genital tract.[9] The diagnosis of genital schistosomiasis can be made by demonstrating the presence of ova on cytological material. Swart and Vander Merwe[10] reported making a diagnosis of genital schistosomiasis on a wet-smear preparation in an outpatient clinic. Our patient had 2 cervical cytological examinations that failed to show any ova, so we considered chronic *intestinal* schistosomiasis to be the most likely cause of her symptoms. The relation between cervical cancer and schistosomal infection is still controversial. The presence of schistosomal ova is associated with epithelial changes that can be regarded as precancerous.[10] In our patient, a cytological examination of the cervix initially showed mild dyskaryosis but spontaneously returned to normal after 6 months. Blood tests useful in the diagnosis of schistosomiasis include schistosomal antibody screening which is strongly positive in active disease. The demonstration of eosinophilia, which suggests a parasitic infection of some form, is present in only 42% of cases of schistosomiasis.[11]

The treatment of choice is a single dose of pyraziquantel, 40 mg/kg of body weight, though some African strains, which are increasingly seen in Europe due to expanding international travel, require 30 mg/kg daily for 3 days.[1] Schistosomiasis may not be clinically apparent until years after the patient has left the endemic areas, and the rarity of this condition may pose diagnostic problems for physicians in European countries. However, early diagnosis is crucial in the management of schistosomiasis in order to prevent serious long-term complications.

In summary, this case illustrates that doctors should be aware of chronic schistosomiasis as one of the differential diagnoses of chronic pelvic pain, particularly in women who have a negative screen for STD and come from or visited areas where schistosomiasis is endemic.

### REFERENCES

1. Gilles HM: Schistosomiasis update. Int J Dermatol 27: 400–401, 1988.
2. Doechring E: Schistosomiasis in childhood. Eur J Paediatr 147:2–9, 1988.
3. Joubert J, Fripp PJ, Hay IT, Davel GH, Van Graan

ESJ: Schistosomiasis of the spinal cord—Underdiagnosed in South Africa? S Afr Med J 77:297–299, 1990.
4. Mohamed AE, Al Karawi MA, Yasawy MI: Schistosomal colonic disease. Gut 31:439–442, 1990.
5. Bac DJ, Teichler MJ, Jonker LC, Van der Merwe CF: Schistosomiasis in ectopic or unusual sites. A report of 5 cases. S Afr Med J 72:717–718, 1987.
6. Cosnett JE, Van Dellen JR: Schistosomiasis (Bilharzia) of the spinal cord: Case reports and clinical profile. Q J Med 61:1131–1139, 1986.
7. Contractor QQ, Benson L, Schulz TB, Contractor TQ, Kasturi N: Duodenal involvement in *Schistosoma mansoni* infection. Gut 29:1011–1012, 1988.
8. Vass ACR, Lucy JJ: Bilharzial granuloma of the fallopian tube. Case report Br J Obstet Gynaecol 89:867, 1982.
9. El Tabbakh G, Hamza MA: Carcinoma of the uterine cervix and schistosomiasis. Int J Gynaecol Obstet 29: 263–268, 1989.
10. Swart PJ, Van der Merwe JV: Wet-smear diagnosis of genital schistosomiasis. S Afr Med J 72:631–632, 1987.
11. Gelgfand M: Schistosomiasis in South Central Africa: A Clinicopathological Study. Cape Town and Johannesburg: The Post Graduate Press, Juta & Co. Ltd., 1950.
